# Correction: González-Rellán et al. Anatomy of the Palmar Region of the Carpus of the Dog. *Animals* 2022, *12*, 1573

**DOI:** 10.3390/ani12162019

**Published:** 2022-08-10

**Authors:** Sonia González-Rellán, Andrés Barreiro, José Manuel Cifuentes, Patricia Fdz-de-Trocóniz

**Affiliations:** 1 Department of Anatomy, Animal Production and Clinical Veterinary Science, University of Santiago de Compostela, 27002 Lugo, Spain; 2 Rof Codina Veterinary University Hospital, 27002 Lugo, Spain

## Error in Figure

In the original publication [1], there was a mistake in Figure 3. In response to a suggestion from one of the reviewers (point 6 of Reviewer #2), the authors modified some figures to add a schematic drawing. After the publication of the article, the authors realized that, in Figure 3, this modification changed the placement of some arrows. We have corrected it, and now, the arrows point to the right structures (as it was initially, before the modification). The corrected Figure 3 appears below. The authors apologize for any inconvenience caused and state that the scientific conclusions are unaffected. This correction was approved by the Academic Editor. The original publication has also been updated.


